# Systemic proteasome inhibition triggers neurodegeneration in a transgenic mouse model expressing human α-synuclein under oligodendrocyte promoter: implications for multiple system atrophy

**DOI:** 10.1007/s00401-012-0977-5

**Published:** 2012-04-11

**Authors:** Nadia Stefanova, Walter A. Kaufmann, Christian Humpel, Werner Poewe, Gregor K. Wenning

**Affiliations:** 1 Division of Neurobiology, Innsbruck Medical University, Innsbruck, Austria; 2 Department of Neurology, Innsbruck Medical University, Innsbruck, Austria; 3 Department of Pharmacology, Innsbruck Medical University, Innsbruck, Austria; 4 Department of Psychiatry and Psychotherapy, Innsbruck Medical University, Innsbruck, Austria; 5 Division of Neurobiology, Department of Neurology, Innsbruck Medical University, Anichstr. 35, 6020 Innsbruck, Austria

**Keywords:** Proteasome inhibition, Synuclein, Neurodegeneration, Parkinsonism, Ultrastructure, Transgenic mouse

## Abstract

Multiple system atrophy (MSA) is a progressive late onset neurodegenerative α-synucleinopathy with unclear pathogenesis. Recent genetic and pathological studies support a central role of α-synuclein (αSYN) in MSA pathogenesis. Oligodendroglial cytoplasmic inclusions of fibrillar αSYN and dysfunction of the ubiquitin–proteasome system are suggestive of proteolytic stress in this disorder. To address the possible pathogenic role of oligodendroglial αSYN accumulation and proteolytic failure in MSA we applied systemic proteasome inhibition (PSI) in transgenic mice with oligodendroglial human αSYN expression and determined the presence of MSA-like neurodegeneration in this model as compared to wild-type mice. PSI induced open field motor disability in transgenic αSYN mice but not in wild-type mice. The motor phenotype corresponded to progressive and selective neuronal loss in the striatonigral and olivopontocerebellar systems of PSI-treated transgenic αSYN mice. In contrast no neurodegeneration was detected in PSI-treated wild-type controls. PSI treatment of transgenic αSYN mice was associated with significant ultrastructural alterations including accumulation of fibrillar human αSYN in the cytoplasm of oligodendroglia, which resulted in myelin disruption and demyelination characterized by increased g-ratio. The oligodendroglial and myelin pathology was accompanied by axonal degeneration evidenced by signs of mitochondrial stress and dysfunctional axonal transport in the affected neurites. In summary, we provide new evidence supporting a primary role of proteolytic failure and suggesting a neurodegenerative pathomechanism related to disturbed oligodendroglial/myelin trophic support in the pathogenesis of MSA.

**Electronic supplementary material:**

The online version of this article (doi:10.1007/s00401-012-0977-5) contains supplementary material, which is available to authorized users.

## Introduction

Multiple system atrophy (MSA) is a sporadic neurodegenerative disorder clinically characterized by parkinsonism, cerebellar, autonomic, and pyramidal dysfunction [37]. The pathogenesis of the disease remains largely unknown. The neuropathology includes various degrees of striatonigral degeneration (SND) and olivopontocerebellar atrophy (OPCA) which define the substrate of atypical parkinsonism and ataxia in MSA. α-Synuclein (αSYN)-positive cytoplasmic inclusions are observed throughout the MSA brain in neurons (like in other α-synucleinopathies) and uniquely in oligodendrocytes (named glial cytoplasmic inclusions (GCIs)) and may contribute to the MSA pattern of neurodegeneration [8, 12, 44]. A major role of αSYN in the pathogenesis of MSA has also been suggested by genome-wide association (GWA) studies revealing association of polymorphisms within the αSYN gene (SNCA) with increased MSA risk [1, 35]. Furthermore, recent evidence indicates that dysfunction of the ubiquitin–proteasome system (UPS) is a common pathogenic mechanism in α-synucleinopathies including Parkinson’s disease (PD), dementia with Lewy bodies, and MSA [5]. Specifically, in MSA brain tissue reduced immunoreactivity for the 20S-α subunit of the core proteasome complex has been shown in dopaminergic nigral neurons [5]. The 20S proteasome subunit gene expression has been found to be downregulated in the pons of MSA patients [20]. These data suggest that proteolytic stress in MSA might result from impaired degradation (proteasome dysfunction) and excess aberrant accumulation of αSYN specifically affecting oligodendroglia by yet unknown mechanisms, which eventually lead to selective and wide-spread multisystem neurodegeneration.

Transgenic overexpression of human αSYN (hαSYN) under the control of oligodendroglial promoters has been classically introduced to model MSA-like glial inclusions [40]. Transgenic mice with oligodendroglial overexpression of hαSYN under the control of proteolipid protein (PLP) promoter feature GCIs of hyperphosphorylated insoluble hαSYN with asymptomatic early neurodegeneration and microgliosis [14, 39, 41]. We sought to enhance proteolytic stress through systemic proteasome inhibition in the PLP-hαSYN transgenic mouse [14] and define possible pathogenic mechanisms related to MSA-like neurodegeneration. For the first time we show ultrastructural mechanisms of neurodegeneration related to PSI-accelerated oligodendroglial αSYN accumulation associated with myelin disruption and axonal degeneration providing novel insights into MSA pathogenesis.

## Materials and methods

### Animals and treatment

The generation and characterization of the PLP-hαSYN mice were previously described [14]. Homozygous transgenic PLP-hαSYN mice were obtained from Prof. Philipp Kahle (Tübingen, Germany) and further bred and maintained in a temperature-controlled specific pathogen free (SPF) room under 12-h light/dark cycle with free access to food and water at the Animal Facility of Innsbruck Medical University. All mice were genotyped by tail clip using PCR for human αSYN with the following primers, fwd: 5′-ATG GAT GTA TTC ATG AAA GG-3′; rev: 5′-TTA GGC TTC AGG TTC GTA G-3′ giving a product size of 450 bp. The oligodendroglial αSYN overexpression was determined by 2′,3′-cyclic nucleotide 3′-phosphodiesterase (CNPase)/αSYN double immunofluorescence as previously shown [14, 41]. All experiments were performed according to Austrian law and with permission from the Federal Ministry of Science and Research, Austria. All efforts were made to minimize the number of animals used and their suffering.

Transgenic mice over 12 months of age and age-matched wild-type controls of the background strain C57Bl/6 were randomized into two treatment groups receiving either proteasome inhibitor (PSI) or vehicle. The PSI groups received proteasome inhibitor I (Z-Ile-Glu(OtBu)-Ala-Leu-CHO), a cell-permeable reversible inhibitor of the chymotrypsin-like activity of the 20S proteasome, provided in 50 mM stock solution in DMSO (Calbiochem, La Jolla, CA). PSI was applied subcutaneously every other day over a period of 2 weeks at a concentration of 5 mg per kilo body weight per day in 1.7 % DMSO in saline as based on previous protocols and data [4, 13, 19, 22, 34, 36, 46]. The vehicle-treated groups received 1.7 % DMSO in saline according to the same time schedule. Subgroups were behaviorally tested and killed 2 and 12 weeks after the first PSI/vehicle injection according to the following protocols.

### Chemicals, sera, and antibodies

Paraformaldehyde, osmium tetroxide, uranyl acetate, and pioloform were obtained from Agar Scientific (Stansted, UK). EM grade glutaraldehyde was purchased from Polysciences (Warrington, PA). Thiopental was from Sandoz (Kundl, Austria). Lead(II) citrate was from Merck (Darmstadt, Germany), picric acid from Fluka (Buchs, Switzerland). Normal goat serum was from Bender (Vienna, Austria), bovine serum albumin from Serva (Heidelberg, Germany). Biotinylated antibodies (horse anti-mouse IgG, goat anti-rat IgG) and Elite avidin–biotinylated horseradish peroxidase complex were from Vector Laboratories (Burlingame, CA). Gold-conjugated immunoglobulins were from British BioCell (Cardiff, UK). The nanogold^®^-conjugated Fab′ fragments and the HQ Silver™ Enhancement Kit were from Nanoprobes (Yaphank NY, USA). All remaining chemicals were from Sigma-Aldrich (Vienna, Austria).

The monoclonal anti-DARPP-32 (1:20,000) was a generous gift from Prof. H. Hemmings (New York, USA), monoclonal anti-TH (1:500) and monoclonal anti-CNPase (clone 11-5B, 1:100) were from Sigma (St. Louis, USA), monoclonal anti-human-α-synuclein (15G7, aa 116-131, 1:10) was generously provided by PJ Kahle (Tübingen, Germany), and monoclonal anti-α-synuclein (clone 42, aa 15-123, 1:50) was from BD Transduction Laboratories (Lexington, KY). Monoclonal anti-mouse glial fibrillary acidic protein (GFAP, 1:100) was from Roche (Vienna, Austria) and monoclonal anti-mouse CD11b (1:150) was from Serotec (Oxford, UK). All antibodies were applied successfully in immunohistochemistry before [15, 30, 32, 38]. The specificity of the immunostainings was verified in the current study by omitting primary antibodies and incubating sections with the full set of secondary antibodies.

For immunoblotting polyclonal anti-ubiquitin antibody was purchased from Abcam (Cambridge, UK, 1:2,000), polyclonal anti-LC3B antibody from Cell Signaling Technology (Beverly, MA, 1:1,000), and monoclonal anti-actin antibody from BD Transduction Laboratories (Franklin Lakes, NJ, 1:10,000).

### Tissue processing

For morphological analysis animals were perfusion fixed under thiopental anesthesia (12 mg/100 g body weight, intraperitoneally; either on day 15 or day 85 after the first PSI/vehicle injection) with 10 ml phosphate buffered saline (PBS; 25 mM, 0.9 % NaCl, pH 7.4) followed by chilled fixative in PBS (buffer conditions are given below for the different techniques used). Brains were rapidly removed from skulls, postfixed overnight in the same fixative at 4 °C, and washed in 0.1 M PBS containing 0.05 % sodium azide at 4 °C.

### Immunohistochemistry for light microscopy

Brains were perfusion fixed with 4 % paraformaldehyde in PBS. Following fixation and dissection, they were cryoprotected in 25 % sucrose in PBS, quickly frozen by immersion in cold (–50 °C) isopentane, and stored at –70 °C. Frozen brains were sliced serially at 40 μm using a cryostat (Leica, Nussloch, Germany). One series of sections per animal was mounted on slides and underwent cresyl violet (CV) staining to define the borders and determine the numbers of neurons in the inferior olivary complex, pontine nuclei, and deep cerebellar nuclei. Series of brain sections underwent immunohistochemical staining according to a standard immunoperoxidase protocol for free-floating sections. Shortly thereafter, endogenous peroxidase activity was quenched in 0.3 % H_2_O_2_ in PBS. After 5 % normal serum blocking in PBS containing 1 % bovine serum albumin (BSA) sections were incubated with the primary antibody overnight at 4 °C, followed by incubation in biotinylated secondary antibody (1:500 for 1 h) at room temperature. After incubation in Vectastain ABC reagent the immunohistochemical reaction was developed with 3,3′-diaminobenzidine (DAB) and sections were mounted onto gelatine-coated slides, dehydrated, and coverslipped with Entellan.

### Ultrastructural analysis

Brains were perfusion fixed with phosphate buffer (PB; 0.1 M, pH 7.4) containing 2 % formaldehyde and 2.5 % glutaraldehyde. Tissue blocks from the substantia nigra, cerebellum, and brain stem were dissected, and coronal sections were sliced at 60 μm with a Vibroslicer (VT1000S; Leica Microsystems, Vienna, Austria). Sections were treated with 2 % osmium tetroxide in PB and embedded in epoxy resin (Durcupan^®^ ACM Fluca). Serial ultrathin sections (70–80 nm) were collected on formvar-coated copper slot grids and contrasted by means of uranyl acetate and lead citrate. Sections were examined in a Philips CM120 transmission electron microscope (TEM), equipped with a Morada CCD camera (Soft Imaging Systems, Muenster, Germany). Whole images were level-adjusted, sharpened, and cropped in Photoshop (Adobe^®^) without changing any specific feature within.

### Pre-embedding electron microscopy

Brains of animals were perfusion fixed with PB containing 4 % formaldehyde and 0.05 % glutaraldehyde. Tissue blocks were dissected and coronal sections at 60 μm were sliced with a Vibroslicer (VT1000S; Leica Microsystems). Immunolabeling for electron microscopy according to the avidin–biotinylated horseradish peroxidase complex (ABC) method was performed as described previously [33]. Briefly, sections were incubated with increasing gradients of sucrose (5, 10, and 20 %) in PB, flash frozen on liquid nitrogen, and rapidly thawed in PB to increase penetration of reagents. Sections were then incubated in 50 mM glycine in Tris-buffered saline (TBS; 50 mM, 0.9 % NaCl, pH 7.4) for 1 h at RT for quenching of free aldehyde groups, followed by incubation in 10 % NGS and 2 % BSA in TBS for 2 h at RT for blocking of nonspecific binding sites. Primary antibodies were then applied for 48 h at 4 °C in TBS containing 2 % BSA at a dilution of 2 μg/ml. After washing in TBS, biotinylated secondary antibodies were applied (1:200 in TBS containing 2 % BSA, for 24 h at 4 °C). Sections were then treated with an avidin–biotinylated horseradish peroxidase complex (Vectastain^®^ ABC; 1:100 in TBS, for 3 h at RT). The sections were reacted with 0.05 % DAB and 0.003 % H_2_O_2_ in Tris buffer (TB; 50 mM, pH 7.4) for 6–7 min at RT, and washed in TB.

Nanogold silver amplification: Alternatively, incubation with primary antibodies was followed by applying nanogold^®^-conjugated Fab′ fragments (1:100 in TBS). Gold particles were amplified with silver for 5–7 min at RT using the HQ Silver™ Enhancement Kit. Sections were treated with 2 % osmium tetroxide in PB, contrasted by means of 1 % uranyl acetate in 50 % ethanol, and embedded in epoxy resin (Durcupan^®^ ACM Fluca). Serial sections (70–80 nm) were collected on formvar-coated copper slot grids. Control experiments included pre-embedding immunoelectron microscopy for αSYN in brain slices of wild-type C57Bl/6 mice or verification of the specificity of the immunostainings by omitting the primary antibody. Sections were examined in a Philips CM120 TEM, equipped with a Morada CCD camera (Soft Imaging Systems). Whole images were level-adjusted, sharpened, and cropped in Photoshop (Adobe^®^) without changing any specific feature within.

### Measurement of proteasome activity

The measurement of proteasome activity was performed as previously described [43]. Brain tissue was homogenized by sonication in ice-cold PBS (pH 7.4) and protein concentration was determined by bicinchoninic acid (BCA) protein assay (Sigma, St. Louis, MO) according to a standard protocol. Brain homogenates (100 μg protein) were incubated in Eppendorf tubes with 100 mM Tris–HCl (pH 7.5), 2 mM CaCl_2_, 1 mM ATP, and 5 μM fluorogenic substrate III (Suc-LLVY-AMC, Calbiochem, Darmstadt, Germany) or substrate IV (Z-VKM-AMC, Calbiochem, Darmstadt, Germany) up to a total volume of 250 μl. The tubes were incubated at 37 °C for 3 h and the fluorescence was counted in black 96-well plate in a Beckman Coulter fluorescence reader at excitation 360 nm and emission 465 nm. Assay buffer without brain extract served as a background control. To control for sensitivity and specificity of the Z-Ile-Glu(OtBu)-Ala-Leu-CHO-induced proteasome inhibition in vitro, 100 μM of Z-Ile-Glu(OtBu)-Ala-Leu-CHO was added to the assay of control mouse brain homogenate (100 μg) as stated above and further reported as “in vitro assay”.

### Immunoblotting

For protein analysis mice were anesthetized with thiopental, perfused intracardially with PBS, and brains were quickly removed, snap frozen in liquid nitrogen, and finally stored at −80 °C. Probes were homogenized on ice in lysis buffer containing Complete Mini Protease Inhibitor Cocktail (Roche Applied Science, Indianapolis, IN), protein concentration was measured by BCA protein assay (Sigma, St. Louis, MO), and aliquots for further analysis were stored at −80 °C. Protein balanced samples (30 μg each) were separated in 10 % SDS–polyacrylamide gels (Novex, San Diego, CA) and electrotransferred to nitrocellulose membranes (Hybond-C, Amersham, Buckinghamshire, UK). Blots were blocked for 1 h with 2 % milk powder in PBS containing 0.05 % Tween-20. Primary antibody incubations were performed overnight at 4 °C followed by 60 min incubation with the appropriate secondary antibody [ECL anti-mouse IgG and anti-rabbit IgG, horseradish peroxidase linked (GE Healthcare, Buckinghamshire, UK)]. Detection with enhanced chemo luminescence reagent (ECL; Amersham Pharmacia Biotech) was used. Films were scanned into tif format using an Epson Perfection 1640SU flatbed scanner, and images were analyzed and quantified with ImageJ software. Relative intensity was determined as the ratio between sample value measured and standard actin value on the same membrane.

### Behavioral tests

#### Open field activity

To test the locomotor activity of the mice we applied the Flex Field Activity System (San Diego Instruments, San Diego, California), which allows monitoring and real-time counting of horizontal and vertical locomotor activity by 544 photo-beam channels. Mice were placed in the center of the open field (40.5 × 40.5 × 36.5 cm) and tested for a 15-min period always at the same time of the day (5:00 p.m.). The tests were performed in a dark room that was completely isolated from external noises and light during the test period. The sum of counts in the horizontal and vertical plane was defined and used for further analysis.

#### Stride length analysis

The stride length was measured applying the DigiGait™ Analysis System (Mouse Specifics, Quincy, MA) as previously described [2]. Mice were placed on a transparent motor-driven treadmill belt and the gait was recorded with a high-speed digital video camera placed below the belt (speed 25 cm/s). The collected images were analyzed with the specific DigiGait Software 9.0 (Mouse Specifics, Quincy, MA) and the mean stride length was determined.

#### Image analysis

Unbiased stereological analysis was performed as previously described [25]. The numbers of dopaminergic neurons in substantia nigra pars compacta (SNc), DARPP-32-positive neurons in striatum and nucleus accumbens, CV-stained neurons in pontine nuclei, deep cerebellar nuclei, and inferior olive were estimated by an optical fractionator (Nikon E-800 microscope, Nikon digital camera DXM 1200; Stereo Investigator Software, MicroBrightField Europe e.K., Magdeburg, Germany). DARPP-32-stained Purkinje cells were counted in a region outlined to include only the Purkinje cell layer [11]. Gliosis was measured by determining mean gray values of GFAP and CD11b immunoreactivity in striatum, corpus callosum, substantia nigra, cerebellum, and brain stem at constant acquisition camera settings with values of 0 for black and 256 for white. Next, optical density (OD) was obtained after a transformation of mean gray values by using the formula OD = −log (mean gray/256) [42].

TEM images of transversely cut, myelinated axons were taken by means of a Morada CCD camera (Soft Imaging Systems) linked to a Philips CM120 TEM at a magnification of ×25,000. The axon diameter was measured using the software iTEM CE (Olympus Soft Imaging Solutions), and the minimal axon diameter of each axonal profile was used for further analysis. The numerical ratio between the diameter of the axon proper and the outer diameter of the myelinated fiber is termed the g-ratio and an increase in the g-ratio reflects a reduced amount of myelination [27]. The g-ratio was determined in SNc and cerebellum of both PSI- and vehicle-treated mice by analyzing 100 axons of approximately 1-μm diameter in each region.

The density of αSYN immunostaining was assessed in oligodendroglia at the level of the soma (*n* = 20 cells per group) using the software iTEM CE (Olympus Soft Imaging Solutions). Sixteen-bit images of the area of interest were acquired by means of a Morada CCD camera (Soft Imaging Systems, Münster, Germany) linked to a Philips CM120 TEM at a magnification of ×11,500 on two consecutive sections per cell. Four gray value measurements of DAB-immunoperoxidase reactions were taken per image along the gray scale and the mean gray value was calculated for each cell. Intensity values representing the “black” (equivalent to gray value 4,600) and “incident” levels (equivalent to gray value 8,500) were calibrated to the images before measurement. The OD was then calculated per cell according to the formula OD = −log (gray value measured/maximum gray value) as done before [16]. The mean OD of αSYN DAB-immunoperoxidase reaction for all cells within a group is finally presented as a percentage of the maximal OD within the calibrated range of OD_min_ = 0.89 (incident light) and OD_max_ = 1.15 (maximum reaction product).

### Statistics

Results are shown as the mean ± SEM, as indicated in each figure. Repeated measures two-way analysis of variance (ANOVA) was used to compare vehicle- and PSI-treated wild-type and transgenic mice 2 and 12 weeks after the first injection. For comparison of g-ratios two-way ANOVA with factors treatment (PSI or vehicle) and region (SNc or cerebellum) was performed. Post-hoc Bonferroni’s test was applied where appropriate. *p* < 0.05 was used to determine statistical significance.

## Results

### Proteasome inhibition in PLP-hαSYN transgenic mice leads to motor impairment

**Fig. 1:**
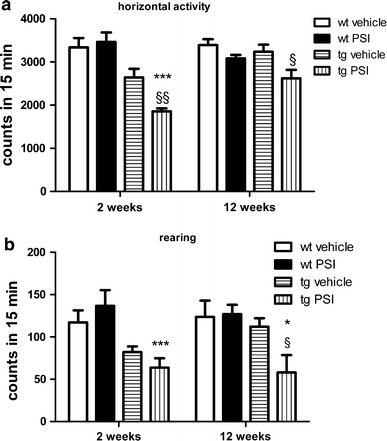
Effects of PSI treatment on motor behavior. **a** Horizontal open field activity showed significant decrease 2 weeks after PSI treatment and remained lower than in vehicle-treated transgenic animals after 12 weeks. **b** Vertical activity (rearing) in an open field arena was still preserved 2 weeks after PSI treatment in tg mice but showed significant decline after 12 weeks as compared to vehicle-treated tg mice. Data, presented here as mean ± SEM, underwent repeated measures two-way ANOVA followed by Bonferroni’s post-hoc test. tg vehicle versus tg PSI: ^§^ *p* < 0.05; ^§§^ *p* < 0.01; wt PSI versus tg PSI: *p < 0.05; ***p < 0.001. *tg* transgenic mice,* wt* wild-type mice

As previously reported transgenic PLP-hαSYN mice showed shortened stride length as compared to age-matched wild-type mice (*p* < 0.01, Supplementary Table S1). DigiGait™ test revealed no further effect of PSI treatment on the stride length of neither wild-type nor transgenic mice as compared to vehicle-treated mice (Supplementary Table S1). Open field analysis detected no effect of PSI treatment in wild-type mice, but showed motor disability measured by reduction of the horizontal activity of PSI-treated compared to vehicle-treated PLP-hαSYN transgenic mice 2 weeks after treatment, which was still observed 12 weeks after treatment (Fig. 1a). Additionally, at the late test point PSI-treated transgenic mice showed significant reduction of rearing behavior (Fig. 1b). PSI-treated transgenic mice showed significantly reduced open field motor activity as compared to PSI-treated wild-type age-matched control mice (Fig. 1a, b).

### Proteasome inhibition in PLP-hαSYN transgenic mice leads to progressive neurodegeneration in selected brain areas

**Fig. 2:**
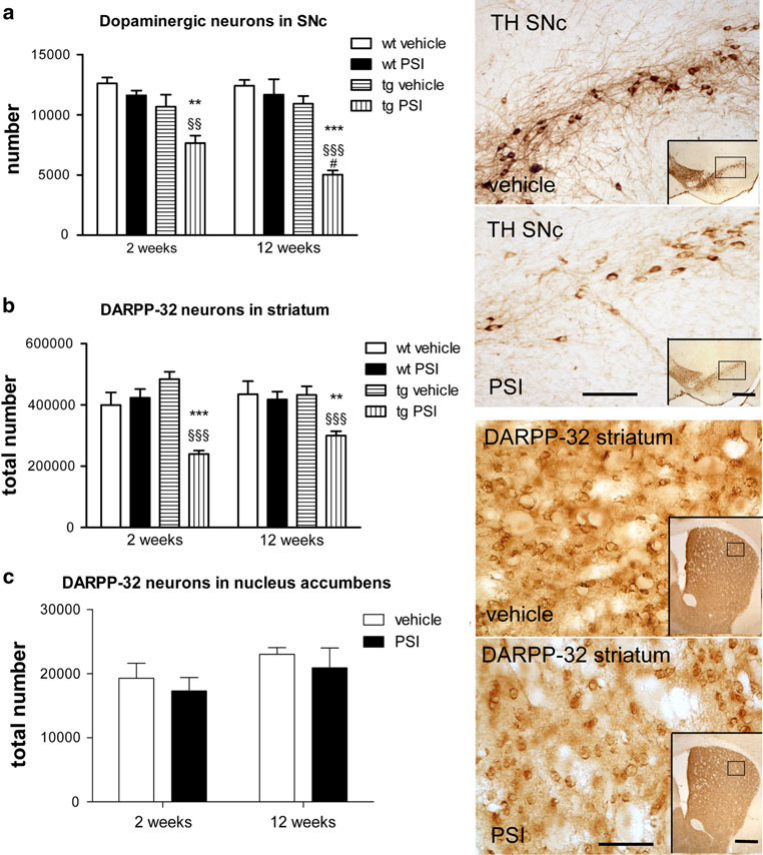
Degeneration in the striatonigral system induced by PSI treatment of PLP-hαSYN mice. **a** The stereologically estimated total number of dopaminergic neurons (detected by TH immunohistochemistry, see *right panel*, *scale bar* 150 μm, *inset scale bar* 500 μm) showed significant decrease in PSI-treated transgenic (*tg*) mice 2 weeks after treatment, which progressed further after 12 weeks**.** No effect of PSI treatment was detected in wild-type (*wt*) mice. **b** Loss of DARPP-32-positive medium spiny neurons in the striatum (see *right panel*, *scale bar* 100 μm, *inset scale bar* 500 μm) was induced by PSI treatment in tg mice as analyzed 2 weeks after treatment and preserved after 12 weeks without further progression of neurodegeneration. No effect of PSI treatment was detected in wt mice. **c** In contrast to striatal DARPP-32-positive neurons, DARPP-32-immunoreactive neurons in nucleus accumbens of tg mice were not affected by PSI treatment at any of the time points studied. Data are presented as mean ± SEM. In all tg groups *n* = 6, in all wt groups *n* = 3. Data were analyzed by repeated measures two-way ANOVA with post-hoc Bonferroni’s test. tg vehicle versus tg PSI: ^§§^ *p* < 0.01; ^§§§^ *p* < 0.001; wt PSI versus tg PSI: **p < 0.01; ***p < 0.001; tg PSI 2 weeks versus tg PSI 12 weeks: ^#^ *p* < 0.05

**Fig. 3:**
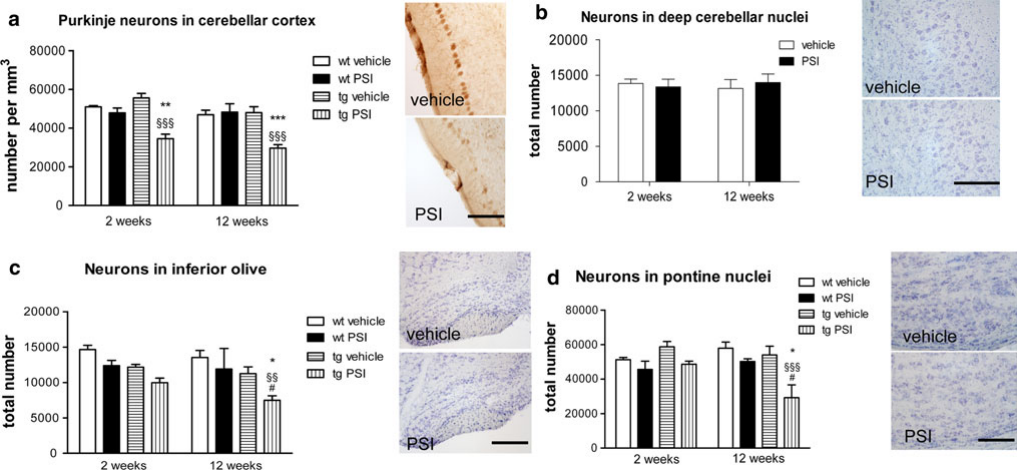
PSI induced degeneration in the olivopontocerebellar system of PLP-hαSYN transgenic mice. **a** Purkinje neurons (visualized by DARPP-32 immunohistochemistry, see *right panel*, *scale bar* 200 μm) showed significant decrease in PSI-treated transgenic (*tg*) mice 2 weeks after treatment, which was further preserved after 12 weeks. No effect of PSI treatment was detected in wild-type (*wt*) mice. **b** No effect of PSI treatment on the number of neurons was detected in the deep cerebellar nuclei of tg mice (analyzed in CV-stained sections, *right panel*, *scale bar* 200 μm). **c** Loss of neurons in the inferior olives of tg mice (see *right panel*, CV staining, *scale bar* 400 μm) was induced by PSI treatment, detectable after 12 weeks, but not after 2 weeks of treatment. No effect of PSI treatment was detected in wt mice. **d** The number of neurons in the pontine nuclei (see *right panel*, CV staining, *scale bar* 200 μm) was decreased upon PSI treatment of tg mice detectable after 12 weeks. No effect of PSI treatment was detected in wt mice. Data are presented as mean ± SEM. In all tg groups *n* = 6, in all wt groups *n* = 3. Data were analyzed by repeated measures two-way ANOVA with post-hoc Bonferroni’s test. tg vehicle versus tg PSI: ^§§^ *p* < 0.01; ^§§§^ *p* < 0.001; wt PSI versus tg PSI: *p < 0.05; **p < 0.01; ***p < 0.001; tg PSI 2 weeks versus tg PSI 12 weeks: ^#^ *p* < 0.05

Systemic PSI treatment of aged wild-type mice had no effect on the number of neurons in any of the regions studied as compared to vehicle-treated animals (Figs. 2, 3). PSI treatment in transgenic mice with oligodendroglial αSYN overexpression induced progressive loss of dopaminergic neurons in SNc compared to vehicle-treated transgenic animals or PSI-treated wild-type age-matched controls (Fig. 2a). PSI-induced loss of dopaminergic neurons in PLP-hαSYN transgenic mice was detectable 2 weeks after treatment (28 %) and progressed up to 54 % neuronal loss at 12 weeks. DARPP-32 positive medium spiny neurons in the transgenic striatum showed significant reduction as a result of PSI treatment detectable 2 weeks and 12 weeks after treatment without progression between weeks 2 and 12 (Fig. 2b). Furthermore, PSI-treated transgenic mice showed significantly lower number of DARPP-32-positive striatal neurons as compared to PSI-treated wild-type mice (Fig. 2b). At the same time DARPP-32 positive neurons in transgenic nucleus accumbens were not affected by the PSI treatment at any of the time points studied (Fig. 2c).

Significant loss of Purkinje cells in the cerebellar cortex was induced early after PSI treatment in transgenic PLP-hαSYN mice and persisted without further progression at 12 weeks after treatment (Fig. 3a). PSI-treated transgenic mice showed significantly reduced numbers of Purkinje neurons as compared to PSI-treated wild-type mice (Fig. 3a). In parallel, there was progressive loss of neurons in the inferior olivary complex (Fig. 3c) and the pontine nuclei in transgenic mice (Fig. 3d), whereas neurons in the deep cerebellar nuclei were not affected by the PSI treatment at any of the time points studied (Fig. 3b).

To define the contribution of gliosis to the observed PSI-induced neurodegeneration in the PLP-hαSYN transgenic mice, the optical density of microglial (CD11b) and astroglial (GFAP) markers was measured in five different brain regions. No significant difference in the optical density of CD11b and GFAP immunostaining was found between vehicle- and PSI-treated transgenic mice, 2 and 12 weeks after treatment (Supplementary Fig. S1).

### Reduced brain proteasome activity by PSI treatment in PLP-hαSYN transgenic mice is associated with high molecular weight ubiquitin accumulation but no significant change of autophagy

**Fig. 4:**
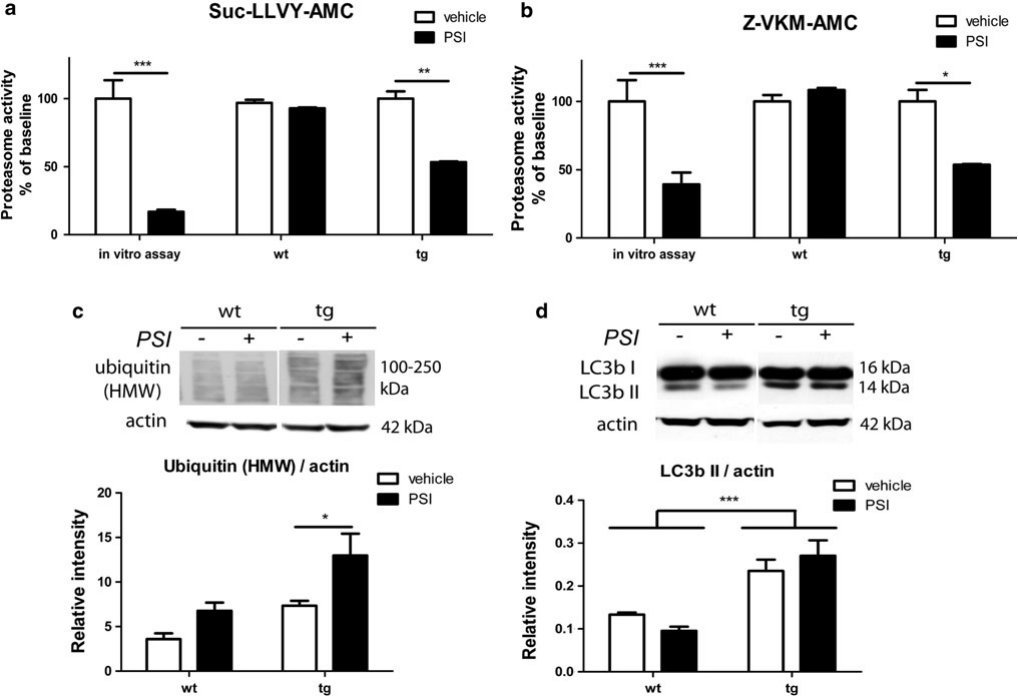
Effects of systemic PSI treatment on the ubiquitin–proteasome system and the autophagy–lysosomal pathway in the brain. **a** Proteasome activity given as a percentage of baseline fluorescence of substrate III (Suc-LLVY-AMC) was reduced by about 93 % after in vitro application of PSI (in vitro assay) and reduced by about 50 % after in vivo application of PSI in transgenic (*tg*) mice at 2 weeks after first intoxication. However, no effect of PSI treatment on brain proteasome activity was measured at the same time point in wild-type (*wt*) mice. **b** Proteasome activity given as a percentage of baseline fluorescence of substrate IV (Z-VKM-AMC) was reduced by about 61 % after in vitro application of PSI (in vitro assay) and reduced by about 50 % after in vivo application of PSI in tg mice at 2 weeks after first intoxication. No effect of in vivo PSI treatment on brain proteasome activity was measured in wt mice at the same time point. **c** Representative immunoblot showing levels of polyubiquitinated high molecular weight (HMW) species as well as densitometric analysis of HMW ubiquitin smear (100–250 kDa) in all mouse groups presented as relative intensity to actin. **d** Representative immunoblot showing levels of LC3B I (16 kDa) and LC3B II (14 kDa) as well as densitometric analysis of LC3B II in all mouse groups presented as relative intensity to actin. Data are presented as mean ± SEM. In all cases *n* = 3. Data were analyzed by two-way ANOVA with post-hoc Bonferroni’s test. *p < 0.05; **p < 0.01; ***p < 0.001

The proteasome activity was significantly reduced in brains of PSI-treated PLP-hαSYN transgenic mice as measured with two different fluorogenic chymotrypsin substrates 2 days after completing the PSI application (Fig. 4a, b). At the same time point PSI treatment had no effect on brain proteasome activity in wild-type mice supporting data from previous studies (Supplementary Table S2). In respect to these results we identified increased accumulation of high molecular weight ubiquitinated protein species in the brains of transgenic but not in wild-type mice treated with PSI (Fig. 4c). Because a potential crosstalk between the two degradation pathways, the ubiquitin–proteasome system and the autophagy–lysosomal pathway, has been suggested, we measured the levels of LC3B II, a classical marker of autophagosome formation. PLP-hαSYN transgenic mice showed significantly increased LC3B II/actin ratios in the brain as compared to wild-type age-matched mice possibly reflecting increased level of autophagy due to transgenic hαSYN overexpression; however, PSI treatment had no further effect on this ratio either in transgenic or in wild-type mice (Fig. 4d). Twelve weeks after systemic treatment with the reversible proteasome inhibitor Z-Ile-Glu(OtBu)-Ala-Leu-CHO no significant changes in the brain proteasome activity were detectable either in PLP-hαSYN transgenic mice (with substrate III: 86.8 ± 1.3 % of baseline; with substrate IV: 91.4 ± 0.8 % of baseline, for all *n* = 3), or in wild-type controls (with substrate III: 116.8 ± 3.4 % of baseline; with substrate IV: 103.6 ± 6.0 % of baseline, for all *n* = 3).

### Ultrastructural analysis of the effects of systemic PSI treatment in the brain of PLP-hαSYN transgenic mice

**Fig. 5:**
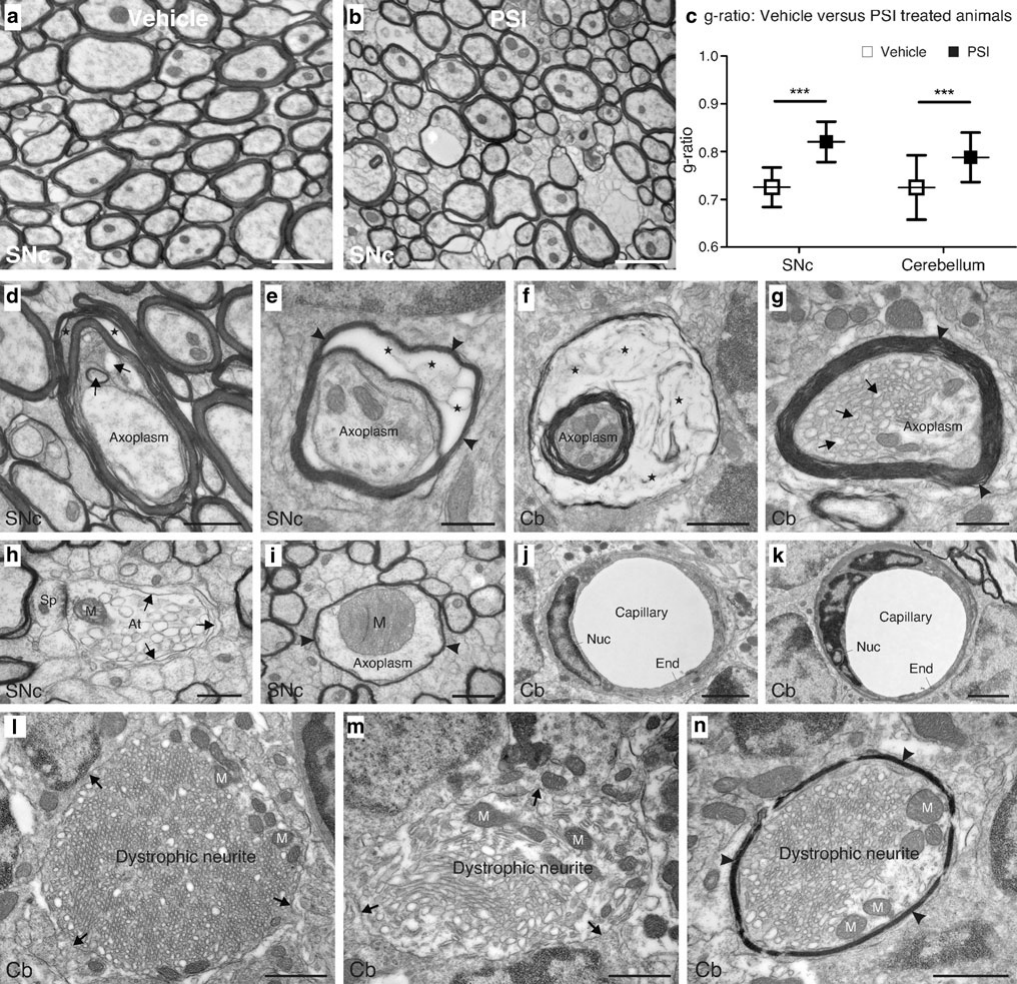
Ultrastructural characterization of the effects of PSI treatment in PLP-hαSYN transgenic mice 12 weeks after first intoxication. **a** In SNc of vehicle-treated animals, signs of degeneration are mild and myelinated axons of regular shape and size are observed. Infrequently, distortions of the axon–glia interface and enlarged extracellular spaces are seen. **b** Upon PSI treatment, numerous signs of tissue damage arise, such as thinning of the myelin sheath, tissue disruption, distortions of the axon–glia interface, and membrane splitting. **c** Calculating the g-ratio confirms thinning of the myelin sheath to a significant extent, both in SNc and the alveus of the cerebellum of PLP-hαSYN transgenic mice upon PSI treatment. **d** In a degenerating axon at an early stage, myelin lamellae are undulated and detached (*asterisks*). The inner tongue is enlarged and shows membranous inclusions and vacuolar profiles (*arrows*). The axoplasm appears normal and intact. **e** In some cases, signs of axonal degeneration are more pronounced with thinned and dilated myelin (*arrowheads*), and split lamellae giving rise to an open space displaying scattered, fibrillary inclusions (*asterisks*). Parts of the axoplasm are darkened and amorphous. **f** At a state of pronounced degeneration, a wide area is formed in between split lamellae, which is occupied by fibrillary inclusions (*asterisks*). **g** In some axons, the myelin sheath appears normal despite mild forms of membrane splitting (*arrowheads*), but the axoplasm is occupied by irregular, tubular profiles to a large extent (*arrows*), possibly representing beginning of axonal swelling. **h** A degenerating axon terminal, synapsing with a small dendritic spine, displays signs of vacuolization and an undulated plasma membrane (*arrows*). **i** The myelin sheath of this axon is thinned and the mitochondrium is unusually enlarged. **j** A capillary in the cerebellum of a control wild-type animal displays regular structure with a normal endothelial cell, basal lamina, and perivascular glia. **k** In comparison, the endothelial wall of a capillary in the cerebellum of a PLP-hαSYN mouse is thinned. **l**, **m** Some non-myelinated dystrophic neurites display abnormal accumulation of tubular and irregular vacuolar profiles within dark amorphous material, accompanied by undulation of the outer membrane (*arrows*). **n** A dystrophic neurite with a thin myelin sheath (*arrowheads*) shows abnormal tubular profiles, and mitochondria are aggregated in the remainder of the cytoplasm. *At* axon terminal, *Cb* cerebellum, *End* endothelial cell, *M* mitochondrium, *Nuc* nucleus, *SNc* substantia nigra pars compacta, *Sp* spine. *Scale bars* 1.5 μm in **a**, **b**, **j**, **k**; 700 nm in **d**, **i**; 500 nm in **e**; 1 μm in **f**, **l**, **m**, **n**; 600 nm in **g**, **h**

In light of the effects of PSI on the motor behavior and the integrity of the striatonigral and olivopontocerebellar system in PLP-hαSYN transgenic mice along with a transient inhibition of the proteasome activity in the brain, we analyzed the ultrastructure of cellular profiles in SNc, cerebellum, and brain stem of PLP-hαSYN transgenic mice 12 weeks after PSI or vehicle treatment. In vehicle-treated PLP-hαSYN transgenic mice, signs of degeneration were mild. Myelinated axons were regular in shape, size, and density, and just infrequently, distortions of the axon–glia interface and unusual enlargements of extracellular space were observed. Otherwise, myelinated axons appeared smooth and intact (Fig. 5a). Following PSI treatment of PLP-hαSYN transgenic mice, numerous signs of tissue damage were observed, ranging from cell disassembly to distortions in the fine structure such as thinning of axonal myelin sheath (Fig. 5b). In order to provide an estimate for the thickness of the myelin sheath, we calculated the g-ratio of myelinated axons in SNc and cerebellum of PLP-hαSYN transgenic mice upon PSI or vehicle treatment. In both areas, the myelin sheath was thinned upon PSI treatment as measured by significant increase in the g-ratio (*p*_SNc_ < 0.001; *p*_cerebellum_ < 0.001; for all *n* = 100; Fig. 5c). Myelinated axons also showed other signs of degeneration in all areas investigated. Mild forms of degeneration included undulated and split myelin lamellae and enlargement of the inner oligodendrocytic tongue with membranous inclusions and irregular vacuolar profiles (Fig. 5d). More pronounced signs of degeneration were dilated myelin sheaths with split, thinned myelin lamellae giving rise to broad areas occupied by fibrillary inclusions. The axoplasm often appeared darkened and amorphous (Fig. 5e). Sometimes, myelin sheaths were so aberrantly dilated that a wide space in between the split myelin lamellae was formed making room for extensive deposition of fibrillary material (Fig. 5f). Other signs of axonal degeneration in axonal fibers were irregular, tubular profiles in the axoplasm (Fig. 5g) and enlarged mitochondria (Fig. 5i). Also at the axon terminals, severe signs of degeneration were observed. These comprised irregular vacuolization and undulation of the axonal plasma membrane (Fig. 5h). Other forms of neuronal degeneration included accumulation of disordered, tubular structures and amorphous, electron dense material between the tubules or irregular vacuoles in non-myelinated (Fig. 5l, m) and myelinated dystrophic neurites (Fig. 5n). Such degenerating neuronal elements were abundant in PSI-treated PLP-hαSYN transgenic mice but rarely seen in vehicle-treated transgenic mice. We also analyzed the morphology of blood vessels in PLP-hαSYN transgenic mice compared to control animals with and without PSI treatment, focusing on capillaries (Fig. 5j, k). We did not see distinct changes in their ultrastructure despite a tendency for thinning of the capillary wall formed by endothelial cells in PLP-hαSYN transgenic mice (Fig. 5k) compared to control animals (Fig. 5j). Nucleus of endothelial cells and subcellular organelles such as mitochondria or Golgi apparatus appeared normal.

**Fig. 6:**
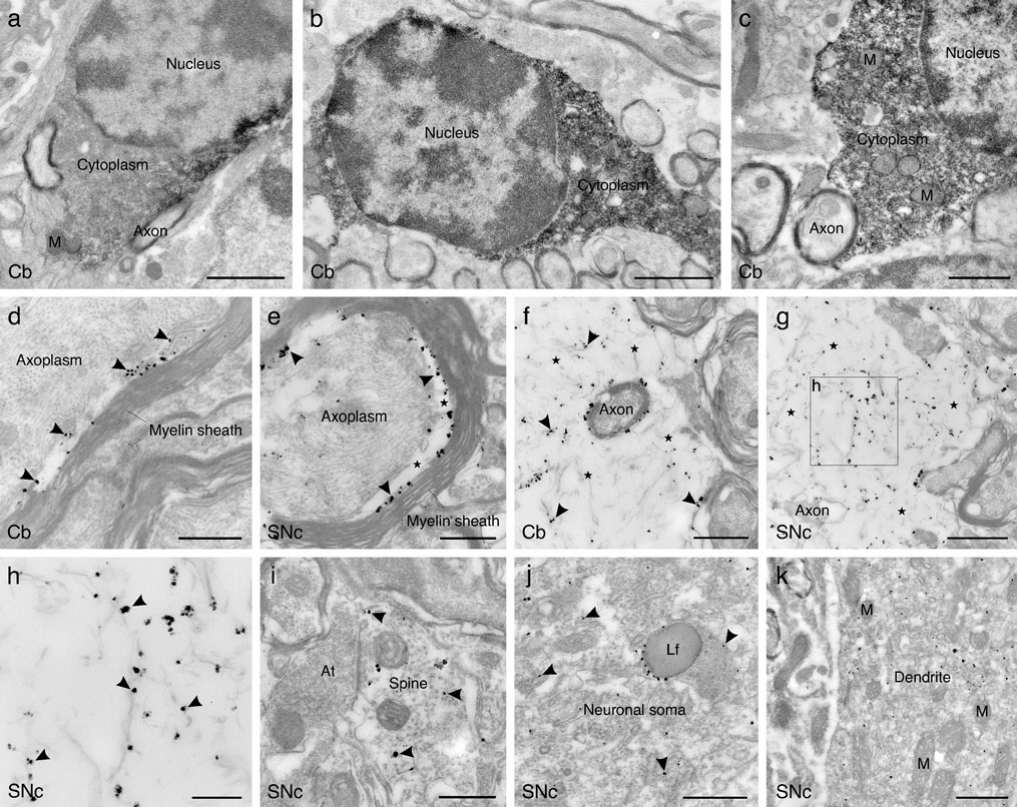
Immunolocalization of transgenic hαSYN and endogenous mouse αSYN by pre-embedding electron microscopy in tissue samples of vehicle- and PSI-treated PLP-hαSYN transgenic mice. **a** In the alveus of the cerebellum of a vehicle-treated animal, the perikaryon of an oligodendrocyte is exclusively labeled for transgenic hαSYN with a 15G7 antibody applying an immunoperoxidase reaction technique, which yields an electron-opaque reaction product. The cytoplasm shows patchy 15G7 immunolabeling, the nucleus and cytoplasmic organelles such as mitochondria are free of immunolabeling. Surrounding axonal and glial profile without signs of degeneration are immunonegative. **b** The same staining pattern is observed in a cerebellar tissue sample of a PSI-treated animal, but the cytoplasm of the oligodendrocyte is uniformly labeled and the density of the immunostaining is markedly enhanced. **c** Subcellular organelles such as mitochondria and the nucleus are immunonegative, shown for a cerebellar oligodendrocyte of a PSI-treated animal at higher magnification. Surrounding neuronal and glial profiles are not labeled. **d** Using an immunometal labeling technique, scattered immunoparticles localizing hαSYN (*arrowheads*) are abundant in the interface between the axoplasm and the myelin sheath in a myelinated axon of the cerebellum of a PSI-treated animal, most likely representing hαSYN localization to the inner tongue of the oligodendrocytic process. Myelin lamellae and the axoplasm are free of any immunolabeling. Surrounding myelinated axons without clear signs of degeneration are immunonegative. **e** The same staining pattern is observed in a myelinated axon in the SNc of a PSI-treated animal. The axon–myelin interface is markedly enlarged and hαSYN immunoparticles (*arrowheads*) are densely packed in this area. **f** In an aspect of a late state degenerating axon in the alveus of the cerebellum of a PSI-treated animal, dense hαSYN immunolabeling of fibrils (*arrowheads*) is apparent in the enlarged area in between split myelin lamellae (*asterisks*). **g** The same localization pattern of hαSYN is observed in an aspect of a late state degenerating axon in the SNc. **h** Higher magnification reveals association of hαSYN immunoparticles (*arrowheads*) with fibrils of 10 ± 0.4 nm in diameter (*n* = 20, measured at a magnification of ×66k in 10 different profiles) loosely arranged in the enlarged area in between split myelin lamellae (*boxed area* in **g**). **i** Scattered immunoparticles localizing endogenous mouse αSYN (*arrowheads*) are observed in the cytoplasm of a dendritic spine in the SNc of PSI-treated transgenic mouse. The axon terminal synapsing with this spine, and neighboring tissue elements are immunonegative. Note: Neuronal αSYN immunoreactivity was detected only with an antibody cross-reacting with mouse αSYN (clone 42), but not with 15G7 antibody detecting hαSYN. **j** Scattered mouse αSYN immunoparticles (*arrowheads*) are observed also in a neuronal soma in the SNc of a PSI-treated transgenic mouse. Immunoparticles are aggregated at a lipofuscin granule. **k** In the same neuron as shown in **j**, the very proximal portion of the principal dendrite is also densely labeled for mouse αSYN. Scattered immunoparticles are seen in the cytoplasm of the dendritic stem, cytoplasmic organelles such as mitochondria and endoplasmic reticulum are immunonegative. *At* axon terminal, *Cb* cerebellum, *Lf* lipofuscin granule, *M* mitochondrium, *SNc* substantia nigra pars compacta. *Scale bar* 1 μm in **a**, **c**; 1.25 μm in **b**; 400 nm in **d**, **e**, **i**; 500 nm in **f**, **j**; 700 nm in **g**, **k**; 200 nm in **h**

Pre-embedding immunoelectron microscopy (IEM) was used then to define the subcellular localization of both transgenic (human) and endogenous (mouse) αSYN in the brains of transgenic MSA mice 12 weeks after treatment with PSI or vehicle. PSI treatment induced increased immunoreactivity for transgenic hαSYN in oligodendroglial cytoplasm in immunoperoxidase labelings (Fig. 6a–c). OD of oligodendroglial αSYN immunoreaction showed about 25 % increase upon PSI treatment (OD_PSI_ 46.06 ± 5.75 % vs. OD_vehicle_ 20.94 ± 6.14 %). Immunometal labeling (nanogold plus silver amplification) for transgenic hαSYN showed different degrees of accumulation of grains at the border between the axolemma and the myelin sheath within the inner oligodendrocyte tongue, up to formation of large inclusions of fibrillar hαSYN (Fig. 6d–h) which were rarely seen in vehicle-treated PLP-hαSYN transgenic mice. Immunolabeled endogenous mouse αSYN (but not hαSYN) was detected accumulating in the cytoplasm of neuronal perikarya and neurites often in association with lysosomes in PSI-treated mice; however, formation of αSYN fibrils and fibrillar inclusions in the neuronal/axonal cytoplasm under PSI treatment was not observed in any of the studied regions (Fig. 6i–k).

## Discussion

Genetic and pathological studies suggest a role of proteolytic stress in MSA [5, 20]. The current study provides experimental evidence for possible mechanisms of progressive MSA-like neurodegeneration resulting from enhanced proteolytic stress related to systemic proteasome inhibition and oligodendroglial α-synucleinopathy. Systemic administration of PSI in PLP-hαSYN transgenic mice featuring GCI-like pathology induced motor disability associated with selective SND and OPCA. Areas uninvolved in the human disease such as deep cerebellar nuclei and nucleus accumbens appeared resistant to PSI treatment in the transgenic mouse model. The PSI-induced neuronal loss in PLP-hαSYN transgenic mice was accompanied by accumulation of high molecular weight ubiquitin conjugates in the brain as well as enhanced hαSYN fibril aggregation in the cytoplasm of oligodendroglial cells up to the inner oligodendrocyte tongue, which led to myelin sheath disruption. Axonal degeneration was associated with signs of mitochondrial stress in the affected neurites as evidenced by enlarged or disrupted mitochondria, as well as dysfunctional axonal transport evidenced by signs of axonal swelling.

Controversial results exist regarding the propensity of systemic proteasome inhibition to induce PD-like nigral degeneration in rats [18, 24, 26, 34, 46]. Systemic application of proteasome inhibitors has failed to cause any detectable behavioral or neuropathological abnormalities relevant to PD in mice [4, 19, 36]. In agreement with these findings we did not observe signs of motor deterioration and neuronal loss in striatonigral and olivopontocerebellar structures upon systemic PSI treatment in wild-type mice. Further, we showed that systemic PSI treatment fails to induce suppression of proteasome activity in the brain of wild-type mice but can reduce transiently up to 50 % the brain proteasome activity in transgenic PLP-hαSYN mice. Differences in the blood–brain barrier permeability between wild-type and transgenic mice may contribute to the observed divergent effects of systemic PSI treatment in wild-type and transgenic mice. At the ultrastructural level, we evidenced normal vasculature with intact endothelial and smooth muscle cells, and regular basal laminae. In case of capillaries, a tendency for thinning of the endothelial wall was observed while endothelial junctions and zonulae occludentes appeared normal. However, physiological and biochemical modifications at the level of endothelial cells and the perivascular glia, such as astrocytic end-feet, might also induce functional alterations of the blood–brain barrier and relate to the overexpression of αSYN but this would need further detailed analysis. Alternatively, the duration of reduction of brain proteasome activity under systemic treatment with the reversible proteasome inhibitor Z-Ile-Glu(OtBu)-Ala-Leu-CHO may differ between wild-type and transgenic mice, which may contribute to the observed differences in the levels of proteasome activity between the genotypes analyzed in this study.

Intriguingly, systemic PSI application in the PLP-hαSYN transgenic mouse resulted in selective SND and OPCA with preservation of neighboring brain structures like nucleus accumbens or deep cerebellar nuclei, which are classically spared also in the human disease, MSA. Although the systemic treatment with the reversible proteasome inhibitor Z-Ile-Glu(OtBu)-Ala-Leu-CHO in PLP-hαSYN transgenic mice had only a transient effect on the brain proteasome activity, neurodegeneration showed progression over an observation period of 3 months with early neuronal loss in SNc, striatum, and cerebellar cortex, which later on extended to the pontine nuclei and the inferior olives as well as being significantly progressed further in the SNc. The neuropathology was reflected by the motor phenotype which was characterized by early disability measured by the open field activity test, providing an early sign of basal ganglia dysfunction. Shortened stride length was found to be an early sign of motor disability in the PLP-hαSYN transgenic mice [38] correlating with the loss of dopaminergic neurons in SNc. The current study confirmed the finding of shortened stride length in PLP-hαSYN transgenic mice versus wild-type aged-matched controls. However, the stride length test failed to show further deterioration as a result of the proteolytic stress in wild-type or PLP-hαSYN transgenic mice over 12 months of age, suggesting that shortening of the stride length may reflect nigral neuronal loss up to a certain degree without registering further progression, indicating a certain limitation of the test.

Because the origin of αSYN in oligodendrocytes in MSA brains is still unclear, the transgenic targeted overexpression of the protein in mouse oligodendroglia therefore provides a mechanistic tool to replicate this critical early event in the disease pathogenesis. At baseline aged PLP-hαSYN transgenic mice show a mild phenotype with (1) insoluble αSYN accumulation in oligodendroglia only rarely forming β-sheet fibrils [14], (2) nigral dopaminergic and brainstem cholinergic neuronal loss [38, 41], and (3) progressive microglial activation [39]. The additional transient exogenous proteolytic stress by systemic application of PSI in these transgenic mice triggers progressive selective neurodegeneration of MSA type (Supplementary Table S3) including SND and OPCA associated with motor deterioration. However, we did not identify further increase of microglial and astroglial activation in the degenerating regions at the time points studied in the PLP-hαSYN transgenic mice. Additional studies may be needed for evaluation of changes in the glial activation profiles. However, one may argue that reactive gliosis and neuroinflammatory response appear temporarily within the period between the two test time points and has not been detected with the used protocol. Alternatively, Middeldorp et al. [28] demonstrate that proteasome inhibitors may reduce GFAP expression in a model of induced astrogliosis in vivo, whereas Elliott et al. [7] provide evidence of the anti-inflammatory properties of proteasome inhibitors. Taken together, these data may suggest an explanation of the lack of further astroglial and microglial activation in the presence of severe PSI-induced neurodegeneration in the PLP-hαSYN transgenic mouse model of MSA. If so, non-inflammatory mechanisms may be the leading pathogenic players of MSA-like neurodegeneration in the PSI-induced transgenic model.

Detailed ultrastructural analysis revealed specific fine structure changes related to the progressive neurodegeneration in PLP-hαSYN transgenic mice following the transient suppression of brain proteasome activity [29]. No Lewy bodies/neuronal fibrillar αSYN inclusions were observed in neurons of the PLP-hαSYN transgenic mice following PSI treatment. However, PSI treatment resulted in disturbed degradation of transgenic αSYN in oligodendroglia. This was associated with increased intracellular accumulation of αSYN in oligodendrocytes as measured by increased OD of αSYN immunoreactivity in oligodendroglial cytoplasm similar to previous in vitro observations [9]. Furthermore, through application of immunometal labeling, accelerated formation of αSYN fibrils of approximately 10-nm width was observed in oligodendrocytes of PSI-treated PLP-hαSYN transgenic mice, reminiscent of the ultrastructure of GCI core fibrils in human MSA [10]. With the applied systemic dose of the reversible proteasome inhibitor Z-Ile-Glu(OtBu)-Ala-Leu-CHO and after 3 months post-intoxication survival, we observed ultrastructural signs of significant oligodendroglial dysfunction associated with enhanced cytoplasmic accumulation of αSYN fibrils rather than signs of oligodendroglial cell death as proposed by earlier in vitro studies in primary PLP-hαSYN oligodendroglia [9]. The increased accumulation of αSYN in oligodendroglial cytoplasm affected the perinuclear soma and the oligodendroglial processes up to the inner tongue opposing the axolemma. The distal accumulation of αSYN fibrils was predominantly associated with myelin sheath disruption and thinning of the myelin (measured here by increased g-ratio), which finally provided compromised myelin support to the affected axons.

Axonal degeneration and mitochondrial stress may follow dysmyelination after proteolytic stress in PLP-hαSYN transgenic mice, similar to recently proposed mechanisms in vitro [17]. However, both myelinated and non-myelinated neurites showed signs of degeneration. This finding may indicate (1) direct, oligodendroglia-independent effects of PSI treatment on neurons, or (2) PSI-induced secondary neurodegeneration due to oligodendroglial dysfunction either related or unrelated to disturbed myelination. Earlier studies have shown that dysfunction of the myelinating glia may lead to axonal swelling and axonal degeneration either uncoupled from the maintenance of compact myelin [21] or associated with defect myelin wrapping [3]. The possible mechanisms of glial support of axons are still under debate [31]; however, oligodendroglial malfunction seems to be the main operative pathogenic mechanism in oligodendroglial α-synucleinopathy enhanced by PSI treatment in the PLP-hαSYN transgenic mice leading to neurodegeneration of MSA type. Finally, the axonal degeneration induced by proteolytic stress in PLP-hαSYN transgenic mice, which may be associated with loss of trophic support and axonal maintenance provided by oligodendroglia/myelin, is reminiscent partly of a model of nonimmune-mediated oligodendrocyte injury [23, 45]. At this stage, the basis of selective vulnerability in MSA remains unclear.

Recent observations have suggested distinct roles and interplay of the ubiquitin–proteasome system and the autophagy–lysosomal pathway in the degradation of αSYN under normal conditions or neuronal overexpression of the protein [6]. Our results suggest a baseline upregulation of autophagy (increased LC3B II signal as a measure of autophagosome formation) in PLP-hαSYN transgenic mouse brains related to the overexpression of αSYN in oligodendroglia in contrast to no change in autophagy rates upon overexpression of αSYN under the regulatory control of the platelet-derived growth factor-β (PDGF-β) neuronal promoter [6]. Furthermore, systemic proteasome inhibition in the PLP-hαSYN transgenic mouse induced accumulation of poliubiquitinated high molecular weight species similar to the one observed by topical application of proteasome inhibitor in PDGF-β-hαSYN transgenic mice. Yet, proteasome inhibition in the presence of oligodendroglial αSYN overexpression had no effect on the rate of autophagy in contrast to previously reported accelerated autophagy upon proteasome inhibition in the presence of neuronal αSYN overexpression [6]. These differences may reflect variable control of both αSYN degradation systems in neurons and oligodendroglia which may need more detailed analysis to identify pathogenic variations relevant to neuronal and oligodendroglial α-synucleinopathies.

In conclusion, the current study provides new evidence supporting the role of oligodendroglial α-synucleinopathy in the pathogenesis of MSA [44] and sheds light on the possible interactions between neurons and dysfunctional oligodendroglial cells that result in a classical non-inflammatory neurodegenerative disorder associated with the pathological accumulation of a misfolded protein.

## Electronic supplementary material

Below is the link to the electronic supplementary material.

Supplementary material 1 (PDF 2,085 kb)

Supplementary material 2 (PDF 61 kb)
