# Supplementary material for: Systemic proteasome inhibition triggers neurodegeneration in a transgenic mouse model expressing human α-synuclein under oligodendrocyte promoter: implications for multiple system atrophy
Source: Acta Neuropathol. 2012 Apr 11;124(1):51–65. doi: 10.1007/s00401-012-0977-5 (PMC3377902; doi:10.1007/s00401-012-0977-5)
Supplement: Supplementary file 1 — Supplementary material 1 (PDF 2,085 kb) [file 401_2012_977_MOESM1_ESM.pdf]

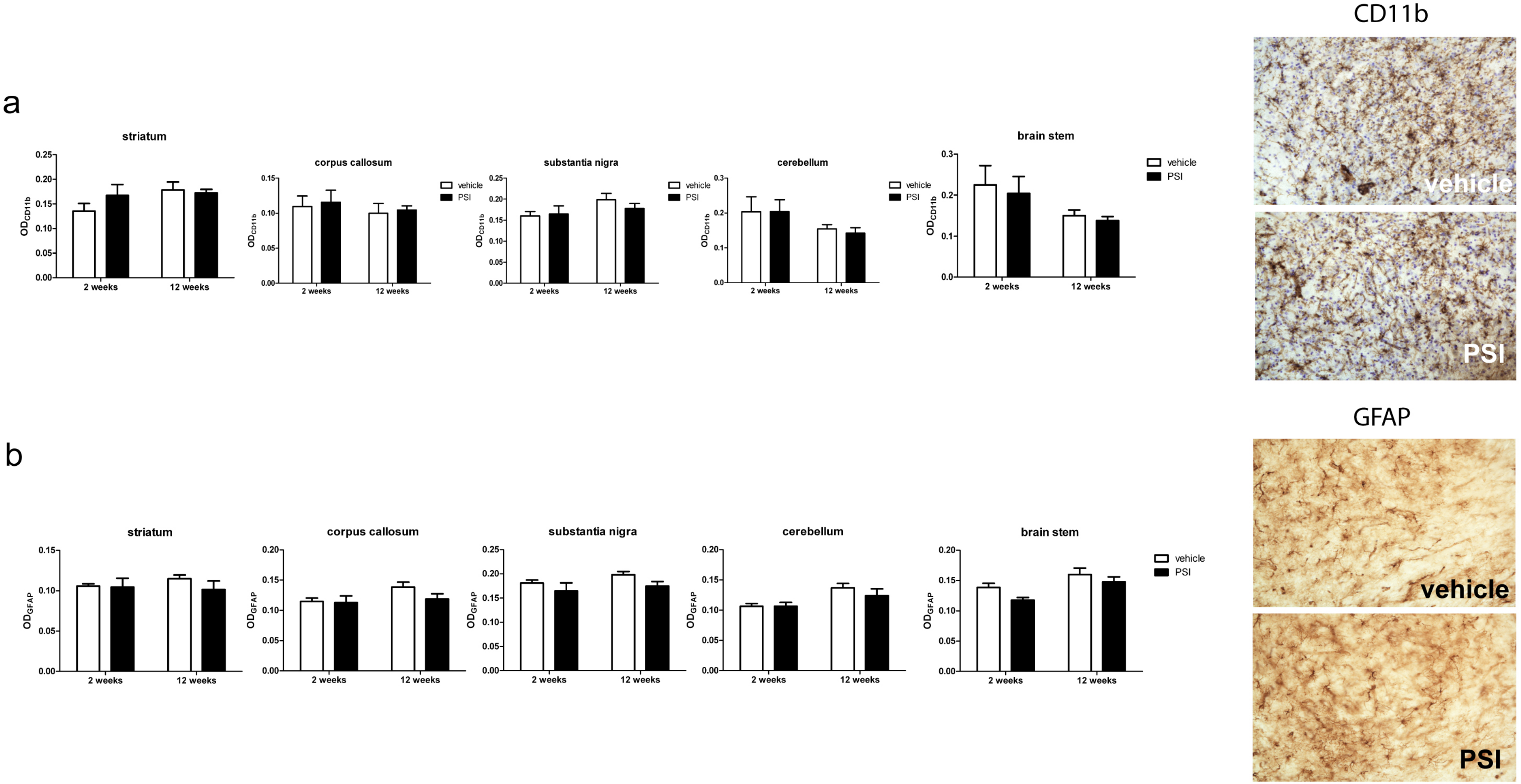

Supplementary Fig. S1. No changes of gliosis were detected in the degenerating regions of PLP-hαSYN transgenic mice upon PSI treatment. (a) Microgliosis was assessed by determining the OD_{CD11b} in the striatum, corpus callosum, substantia nigra, cerebellum and brain stem of vehicle and PSI treated transgenic mice, 2 and 12 weeks after treatment. The photomicrographs in the right panel demonstrate CD11b-positive activated microglia profiles in the cerebellum of both vehicle- and PSI-treated PLP-hαSYN transgenic mice. **(b)** Astrogliosis was assessed by determining the OD_{GFAP} in the striatum, corpus callosum, substantia nigra, cerebellum and brain stem of vehicle and PSI treated transgenic mice, 2 and 12 weeks after treatment. The photomicrographs in the right panel demonstrate GFAP-positive astroglial profiles in the substantia nigra of both vehicle and PSI treated PLP-hαSYN transgenic mice. Data are presented as mean±s.e.m. In all groups n=6. Data were analyzed by two-way ANOVA with post hoc Bonferroni's test. No significant differences between the groups were observed.
