# Supplementary material for: Systemic proteasome inhibition triggers neurodegeneration in a transgenic mouse model expressing human α-synuclein under oligodendrocyte promoter: implications for multiple system atrophy
Source: Acta Neuropathol. 2012 Apr 11;124(1):51–65. doi: 10.1007/s00401-012-0977-5 (PMC3377902; doi:10.1007/s00401-012-0977-5)
Supplement: Supplementary file 2 — Supplementary material 2 (PDF 61 kb) [file 401_2012_977_MOESM2_ESM.pdf]

Supplementary Table S1. Stride length analysis.

Stride length (cm)	2 weeks after treatment			12 weeks after treatment		
	Vehicle	*PSI*	*Effect of treatment (Bonferroni post hoc)*	*Vehicle*	*PSI*	*Effect of treatment (Bonferroni post hoc)*
**tg left hindlimb**	5.185±0.23*** *(n=6)*	4.907±0.23*** *(n=6)*	t=0.69, p>0.05	4.768±0.29*** *(n=6)*	4.906±0.37 *(n=6)*	t=0.34, p>0.05
**tg right hindlimb**	5.145±0.18** *(n=6)*	4.825±0.22*** *(n=6)*	t=0.87, p>0.05	4.596±0.26** *(n=6)*	4.890±0.35** *(n=6)*	t=0.80, p>0.05
**wt left hindlimb**	6.8±0.14 *(n=6)*	6.5±0.23 *(n=6)*	t=0.98, p<0.05	6.5±0.06 *(n=3)*	6.0±0.17 *(n=3)*	t=2.74 p=0.05
**wt right hindlimb**	6.4±0.19 *(n=6)*	6.5±0.16 *(n=6)*	t=0.61, p<0.05	6.3±0.06 *(n=3)*	6.1±0.13 *(n=3)*	t=0.50, p<0.05

tg, transgenic; wt, wild type; Comparison of the respective stride length of wt and tg mice with the same type of treatment, **p<0.01; ***p<0.001.

Supplementary Table S2. Specific effects of systemic proteasome inhibition in wild type versus PLP-hαSYN transgenic mice.

Mouse strain	Motor disability induced by PSI but not vehicle	Neuronal loss induced by PSI but not vehicle	Lewy body-like aggregates in neurons induced by PSI but not vehicle	Oligodendro-glial dysfunction / myelin disruption induced by PSI but not vehicle	Reduced brain proteasome activity	References
C57BL/6	-	-	-	-	-	[4;13;19] and current study
PLP-hαSYN	+	+	-	+	+	current study

Supplementary Table S3. Relevance of the PSI-induced PLP-hαSYN transgenic mouse model to the human MSA pathology.

**Human MSA Pathology**	***PLP-hαSYN***	***PLP-hαSYN+PSI***
GCIs<br>• Hyperphosphorylated insoluble αSYN<br>• αSYN fibril aggregates in oligodendroglia<br>• Polyubiquitination	GCIs<br>• Hyperphosphorylated insoluble αSYN<br>• αSYN non-fibrillar aggregates in oligodendroglia	GCIs<br>• Hyperphosphorylated insoluble αSYN<br>• αSYN fibril aggregates in oligodendroglia<br>• Polyubiquitination
Neuronal αSYN inclusions (cytoplasmic/nuclear)	-	-
Striatonigral degeneration	Nigral degeneration	Striatonigral degeneration
Olivopontocerebellar atrophy	-	Olivopontocerebellar atrophy
Microglial activation	Microglial activation accompanying GCI pathology	Microglial activation accompanying GCI pathology, but not accelerated by PSI treatment
Astrogliosis	-	-
